# Supplementary material for: Evaluation of tarsal injuries in C57BL/6J male mice
Source: PLoS One. 2023 Jun 26;18(6):e0287204. doi: 10.1371/journal.pone.0287204 (PMC10292699; doi:10.1371/journal.pone.0287204)
Supplement: S1 Table — (PDF) [file pone.0287204.s001.pdf]

Supplemental 1 Table

List of Excluded Pathogens	
Viruses	
Ectromelia Virus	Pneumonia virus of mice
GDVII virus	Polyoma virus
Hantaan virus	Reovirus 3
K virus	Rotavirus
LDH elevating virus	Sendai virus
Lymphocytic choriomeningitis	Mouse minute virus
Mouse adenovirus	Mouse norovirus
Mouse cytomegalovirus	Mouse parvovirus
Murine chapparvovirus	Mouse thymic virus
Mouse hepatitis virus	
Bacteria and Mycoplasma	
Bordetella spp	*Filocaterium rodentium*
Citrobacter rodentium	*Mycoplasma pulmonis*
Clostridium piliforme	*Mycoplasma spp.*
Corybacterium bovis	*Salmonells spp.*
Corynebacterium kutscheri	*Streptobacillus moniliformis*
Parasites	
Encephalitozoon cuniculi	Follicle mites
Ectoparasites (fleas, lice, mites)	Protozoa (Giardia, Spironucleus, etc)
Endoparasites (tapeworms, pinworms, helminths)	*Toxoplasma gondii*
Opportunistic Organisms Monitored	
Dematophytes	*Pseudomonas aeruginosa*
Helicobacter spp.	*Staphylococcus aureus*
Klebsiella pneumoniae	*Streptococcus pneumoniae*
Klebsiella oxytoca	Beta-hemolytic Streptococcus spp. (non-group D)
Pasteurella multocida	Trichomonads
Rodentibacter pneumotropicus	*Yersinia enterocolitica*
Pneumocystis murina	*Yersinia pseudotuberculosis*
Proteus mirabilis	
