# Supplementary figures and images for: Evaluation of tarsal injuries in C57BL/6J male mice
Source: PLoS One. 2023 Jun 26;18(6):e0287204. doi: 10.1371/journal.pone.0287204 (PMC10292699; doi:10.1371/journal.pone.0287204)

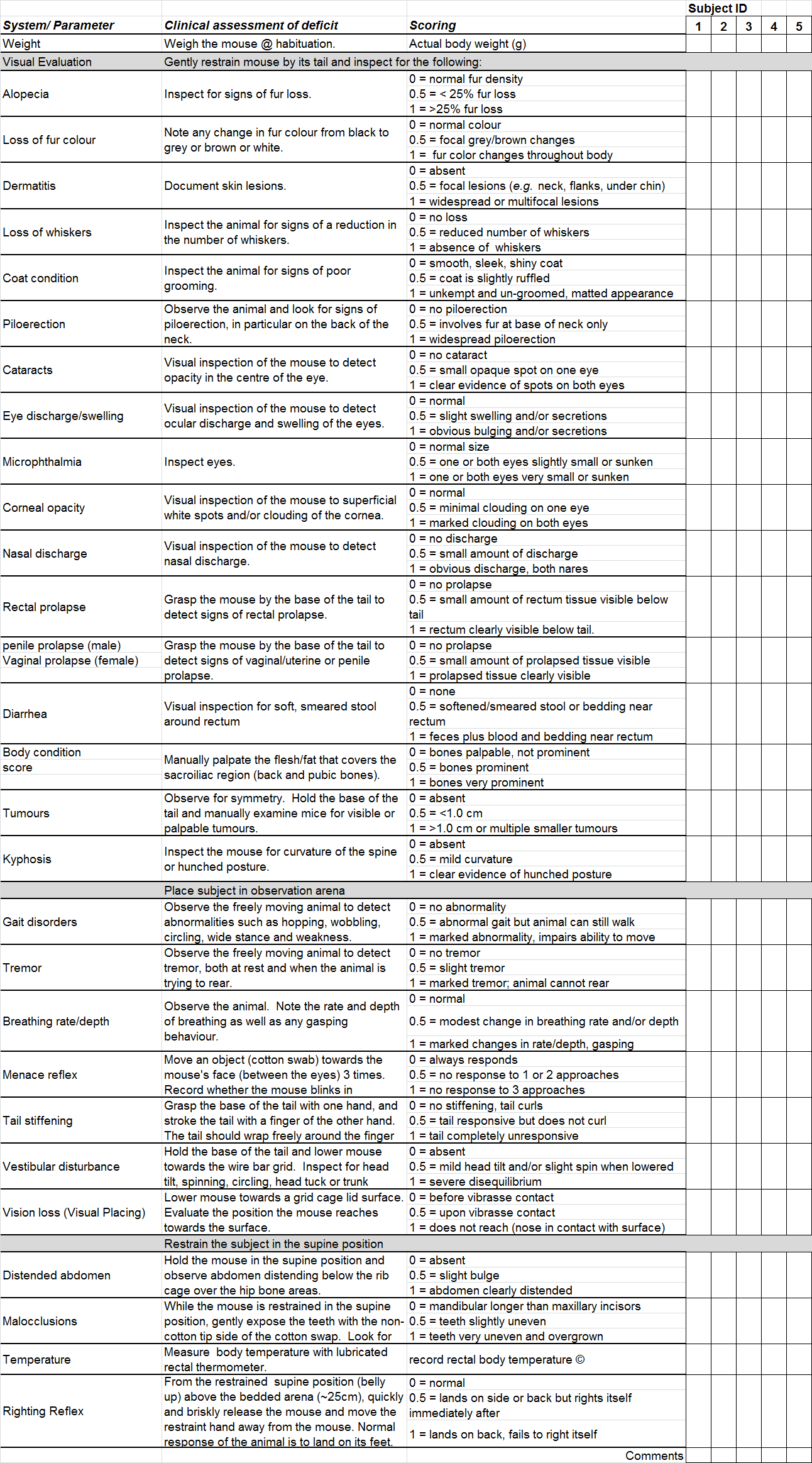

Supplement: S2 Table — (DOCX) [file pone.0287204.s002.docx]
